# TECHNOLOGY AND TRADITIONAL TOOLS FOR INTERGENERATIONAL CONNECTIONS IN HIGHER EDUCATION

**DOI:** 10.1093/geroni/igae098.0701

**Published:** 2024-12-31

**Authors:** Janelle Fassi, Elizabeth Rickenbach, Tina Newsham

**Affiliations:** Chair; Co-Chair; Discussant

## Abstract

While the benefits of connecting older adults with college-aged students are well-documented in literature, the practical application of connecting these individuals together in intergenerational learning spaces is more challenging. This session focuses on the challenges and strategies for successfully implementing and sustaining intergenerational connections in higher education using technology or traditional tools like board games. Each presentation outlines an innovative way for connecting college-aged students with older adults both inside and outside the classroom. The first presentation focuses on the factors that impact the implementation and sustainability of the Cyber-Seniors program through an implementation science framework. The second presentation describes the use of a waitlist control design for examining and identifying the strengths of the Cyber-Seniors Program. The third presentation identifies the feasibility and reflections from an in-person intergenerational game collaboration with Saint Anselm College undergraduate students and community partners. The fourth presentation examines the benefits and challenges of Tellegacy, an intergenerational story-telling project facilitated by university students via in-person, phone or virtual one-on-one visits with older adults in retirement communities. Aligning with the Annual Scientific Meeting 2024 theme, these presentations demonstrate fortitude in refining processes to ensure we are building optimal experiences to connect students and older adults.

